# Genomic and phenotypic analysis of *BRCA2 *mutated breast cancers reveals co-occurring changes linked to progression

**DOI:** 10.1186/bcr3020

**Published:** 2011-09-29

**Authors:** Olafur A Stefansson, Jon G Jonasson, Kristrun Olafsdottir, Hordur Bjarnason, Oskar Th Johannsson, Sigridur K Bodvarsdottir, Sigridur Valgeirsdottir, Jorunn E Eyfjord

**Affiliations:** 1 Cancer Research Laboratory, Faculty of Medicine, University of Iceland, Vatnsmyrarvegur 16, Reykjavik, Iceland; 2 Department of Pathology, Landspitali University Hospital, Hringbraut, Reykjavik, 101, Iceland; 3 Department of Oncology, Landspitali University Hospital, Hringbraut, Reykjavik, 101, Iceland; 4 The Icelandic Cancer Registry, Skogarhlid 8, Reykjavik, 101, Iceland; 5 Roche NimbleGen, Inc., Vínlandsleið 2-4, Reykjavik, 113, Iceland

## Abstract

**Background:**

Inherited mutations in the *BRCA2 *gene greatly increase the risk of developing breast cancer. Consistent with an important role for *BRCA2 *in error-free DNA repair, complex genomic changes are frequently observed in tumors derived from *BRCA2 *mutation carriers. Here, we explore the impact of DNA copy-number changes in *BRCA2 *tumors with respect to phenotype and clinical staging of the disease.

**Methods:**

Breast tumors (*n *= 33) derived from *BRCA2 *999del5 mutation carriers were examined in terms of copy-number changes with high-resolution aCGH (array comparative genomic hybridization) containing 385 thousand probes (about one for each 7 kbp) and expression of phenotypic markers on TMAs (tissue microarrays). The data were examined with respect to clinical parameters including TNM staging, histologic grade, S phase, and ploidy.

**Results:**

Tumors from *BRCA2 *carriers of luminal and basal/triple-negative phenotypes (TNPs) differ with respect to patterns of DNA copy-number changes. The basal/TNP subtype was characterized by lack of pRb (RB1) coupled with high/intense expression of p16 (CDKN2A) gene products. We found increased proportions of Ki-67-positive cells to be significantly associated with loss of the wild-type (wt) *BRCA2 *allele in luminal types, whereas *BRCA2*wt loss was less frequent in *BRCA2 *tumors displaying basal/TNP phenotypes. Furthermore, we show that deletions at 13q13.1, involving the *BRCA2*wt allele, represents a part of a larger network of co-occurring genetic changes, including deletions at 6q22.32-q22.33, 11q14.2-q24.1, and gains at 17q24.1. Importantly, copy-number changes at these *BRCA2*-linked networking regions coincide with those associated with advanced progression, involving the capacity to metastasize to the nodes or more-distant sites at diagnosis.

**Conclusions:**

The results presented here demonstrate divergent paths of tumor evolution in *BRCA2 *carriers and that deletion of the wild-type *BRCA2 *allele, together with co-occurring changes at 6 q, 11 q, and 17 q, are important events in progression toward advanced disease.

## Introduction

Germline mutations in one allele of the *BRCA2 *tumor-suppressor gene confer greatly increased risk of developing breast cancer [1]. The *BRCA2 *gene is known to be involved in error-free DNA repair of double-strand breaks (DSBs) through homologous recombination (HR) [2]. Defects in this mechanism lead to repair of DSBs by error-prone nonhomologous end joining (NHEJ) or single-strand annealing (SSA). The use of error-prone mechanisms for DSB repair can result in genetic changes involving rearrangements and sequence modification at the damaged sites [3]. In this relation, it has been shown that chromosomal instability arises after inactivation of murine *Brca2 *gene, which is in agreement with complex chromosomal changes observed in human breast tumors derived from *BRCA2 *carriers [4,5]. Consistent with Knudson's two-hit hypothesis for tumor suppression, loss of the wild-type (wt) *BRCA2 *allele has been described as an essential event in tumor initiation in *BRCA2 *carriers [6]. This notion has, however, recently been questioned with data demonstrating that loss of *Brca2 *heterozygosity is not a required event in murine models of familial pancreatic cancer and, for human carriers of the *BRCA2 999del5 *mutation, that loss of heterozygosity (LOH) might not be needed to promote carcinogenesis in the pancreas [7]. In addition, breast cancers arising in *BRCA1 *and *BRCA2 *mutation carriers are heterogenic for LOH, suggesting haploinsufficient effects in pathogenesis [8].

Genome-wide expression analyses of breast cancers have led to the identification of biologically and clinically relevant subtypes (that is, luminal-A (LumA), luminal-B (LumB), basal-like, HER2, and normal-like) [9]. The identified subtypes have shown substantial differences with respect to copy-number changes, suggesting that their development adheres to changes in distinct sets of genes, leading to diverging tumor-evolution pathways [10,11]. Of these, the luminal subtypes comprise the majority of all cases (60% to 70%), both characterized by expression of GATA3-regulated genes. The LumB subtype differs from LumA by high expression of proliferation-associated genes with increased levels of MKI67 (Ki-67) as a prominent feature, and are associated with less-favorable disease outcome [12]. Basal-like breast cancers are characterized by expression of genes that pertain to the basal layer of cells in normal mammary glands, such as CK5/6 and CK14. The basal-like and HER2 subtypes are both highly proliferative, poorly differentiated, and, in concordance, show the least-favorable disease outcome [13]. Tumors arising in *BRCA2 *germline mutation carriers mostly display luminal phenotypes of high histologic grade and have prominent expression of proliferation markers, suggesting similarities to the LumB subtype [14]. Relatively few *BRCA2 *tumors have been profiled for copy-number changes, and have mostly been studied by using low-resolution array design, resulting in relatively crude patterns and lack of clarity in identification of key drivers. In our previous report, we found that *BRCA2 *tumors often show highly complex genomes coupled with a unique pattern of copy-number changes [15]. To follow up on these results, we have studied copy-number changes in a larger number of *BRCA2 *tumors by high-resolution array-comparative hybridization (aCGH) microarrays containing 385 thousand probes (about one for each 7 kbp) in breast tumors derived from *BRCA2 *germline mutation carriers (*n *= 33) combined with analysis of phenotypic markers by immunohistochemistry (IHC) on tissue microarrays (TMAs). The purpose of the study was to characterize the spectrum of copy-number changes in tumors obtained from *BRCA2 *carriers with respect to phenotype and clinical parameters.

## Materials and methods

### Study group

Tumor DNA samples (*n *= 33) were derived from patients carrying the *999del5 BRCA2 *germline mutation [16]. The tumors were macroscopically examined by a pathologist to identify portions with invasive tumor cells. DNA extraction was performed on freshly frozen tumor portions by following protocols based on standard procedures by using phenol-chloroform and proteinase K. Clinical parameters, including TNM staging and histologic grade, were obtained from the Department of Pathology, Landspitali University, and are given in Additional File 1. Data on S phase and ploidy, obtained with flow cytometry, were available for 30 of the 33 breast tumors analyzed in this study. Data on patient relapse, that is, events of recurrences or metastases after diagnosis and treatment, were obtained from Landspitali University Hospital. This work was carried out according to permits from the Icelandic Data Protection Commission (2006050307) and Bioethics Committee (VSNb2006050001/03-16). Informed consent was obtained from all patients.

### Array-comparative genomic hybridization

Copy-number changes were studied with high-resolution 385 K oligonucleotide CGH microarrays (Roche NimbleGen, Inc., Reykjavik, Iceland) containing 385 thousand probes covering the human genome with about one probe for each 7 kbp. DNA derived from tumor- and normal blood samples from the same individual were Cy3 and Cy5 labeled, respectively. Sample labeling and hybridizations were carried out according to protocols developed by the manufacturer (NimbleGen Arrays User's Guide-CGH Analysis; Roche NimbleGen, Inc., Reykjavik, Iceland). The aCGH data are available online from ArrayExpress [17] under the accession number E-TABM-1199.

Fine-tiling aCGH analysis was performed by using the 385 K oligonucleotide CGH microarrays covering chromosome 6 with about one probe for each 400 bp (Roche NimbleGen, Inc.), following the manufacturers protocols for sample labeling and hybridization (Roche NimbleGen).

### Tissue microarrays and expression analysis by immunohistochemistry

TMAs construction and their application in expression analysis by immunohistochemistry (IHC) have been described previously [15]. Tumors were assigned to molecular subtypes by using the five-biomarker scheme described in Cheang *et al. *[13] by expression of estrogen receptor (ER), progesterone receptor (PR), human epidermal growth factor receptor 2 (HER-2), epidermal growth factor receptor (EGFR), and cytokeratin 5/6 (CK5/6). Expression of ER and PR were scored positive (IHC score ≥ 1^+^) when IHC staining was observed in more than 1% of tumor cell nuclei. HER-2 positivity was scored for strong membranous staining (IHC score 3^+^), whereas EGFR was scored positive for any, weak or strong, membranous staining (IHC score ≥ 1^+^). Expression of CK5/6 was defined as positive when cytoplasmic and/or membranous staining was observed and otherwise considered negative. Hormone-receptor-positive tumors, those positive for either ER or PR, were assigned to the luminal subtype. Tumors negative for ER, PR, and HER-2 were categorized as triple-negative phenotype (TNP), whereas ER- and PR-negative tumors displaying HER-2 positivity (IHC 3^+^) were assigned to the HER2 subtype. Basal-like phenotype was assigned to triple-negative tumors displaying expression of either EGFR or CK5/6. Estimates for the expression of Ki-67 (MKI67), cyclin-D1 (CCND1), and topoisomerase II alpha (TOP2A) gene products represent counts for the proportion of positive tumor cells, based on nuclear expression, reported on a scale from 0.0 to 1.0 within intervals of 0.05. Luminal tumors showing Ki-67 expression ≥ 0.14 were assigned to the luminal B subtype, whereas the remaining part was considered luminal A [12]. Expression of p16 (CDKN2A) and pRb (RB1) was scored on a discontinuous scale based on counts for the proportion of tumor cells showing nuclear expression (that is, 0 (< 10%), 1^+ ^(10% to 25%), 2^+ ^(25% to 50%), and 3^+ ^(> 50%). The antibodies and dilutions used in IHC expression analysis are listed in Additional File 2.

### TaqMan *BRCA2 *allele-specific quantitative PCR

The proportion of *BRCA2 *wild-type (wt) alleles was quantitatively measured relative to *999del5 BRCA2 *mutated alleles in tumor DNA samples by quantitative polymerase chain reaction (PCR) (7500 Realtime PCR system; Applied Biosystems) with a TaqMan method by using a single *BRCA2*-specific, minor groove-binding probe (MGB-probe) 5'-end-labeled with FAM and a nonfluorescent quencher (NFQ) at the 3' end, a single *BRCA2*-specific forward primer, and two allele-specific reverse primers. Therefore, the PCR for wt and mutant alleles was performed in separate wells. The qPCR assay primers and TaqMan-MGB probe were as follows: Forward primer: 5'-CATGATGAAAGTCGTAAGAAA-3', Reverse primer (mut): 5'-CATGACTTGCAGCTTCTCTTTGTG-3', Reverse primer (wt): 5'-CATGACTTGCAGCTTCTCTTTGAT-3', TaqMan-MGB probe: 5'-TTTATCGCTTCTGTGACA-3'. Reactions were at a 12-μl final volume per well, each containing: TaqMan-MGB at 50 n*M*, forward and reverse primers at 0.5 n*M *each, 6 μl TaqMan Universal PCR Master Mix (Applied Biosystems, Pn. 4304437), and 0.25 μg of sample DNA, with H_2_O to final volume. The PCR: Step(S)1: 50°C for 2 minutes, S2: 95°C for 10 minutes, S3: 95°C for 15 seconds, S4: 60°C for 1 minute, with S3 and S4 were repeated for 40 cycles. The *BRCA2 *wt- to mutant-allele ratios were quantified by measuring differences in fluorescence intensity of FAM performed in duplicate, and the Ct values (number of cycles to reach intensity threshold) were averaged. Ct measures with standard deviation higher than 0.5 (SD > 0.5) were rejected and remeasured. The wild-type to mutant-allele ratios were calculated to wt/allele frequencies by the following equation:

$$\text{Frequency of allel}{\text{e}}_{1}=1∕\left(2^{\text{Δ}\text{Ct}}+1\right)$$

where ΔCt = (Ct of allele_1 _- Ct of allele_2_) [18].

### Statistical analysis and data mining of aCGH data

Preprocessing and segmentation of the aCGH data were carried out as described previously [15]. Normalized log_2 _ratios were replaced with corresponding segment means for each probe represented on the arrays to derive a segmented profile for each tumor. These data were used as input for SAMr (significance analysis of microarrays for R) in two-class or quantitative analysis, in which repeated permutations are used to address multiple testing [19].

Genetic networks represent a set of associated copy-number changes (links) between distinct regions (nodes) of the genome. Here, the average of segmented aCGH log_2 _ratios was computed for each of 811 cytobands with the nucleotide cytoband-locations downloaded by using the UCSC table browser [20]. The pair-wise cytoband-cytoband associations were then determined by hypothesis testing for Spearman's rho (Stats package in R). The *P *values for each of these comparisons were then adjusted for multiple testing by the Holm procedure (Multitest package in Bioconductor for R). Links were then established for associations with *P*_adj _< 0.001, while also having minimum event frequency of 25%. The associations, or "genetic links," represent nonrandom patterns of co-occurring copy-number changes. We then split the links into sets of inter- and intrachromosomal links involving associations between distinct chromosomes (inter), or associations between distinct arms on the same chromosome (intra), respectively. The resulting networks of co-occurring genetic changes were visualized as undirected node-and-edge graphs by making use of the Rgraphviz package in Bioconductor for R.

## Results

### Breast cancer subtypes in *BRCA2 *mutated tumors

Thirty-three female breast cancer patients carrying the same 999del5 *BRCA2 *germline mutation constituted the study group analyzed with array CGH (comparative genomic hybridization). The year of diagnosis was between 1989 and 1999, with the mean age of 50 years (standard deviation (SD) = 12.2) (Additional File 1). Of the 33 tumors analyzed with aCGH, 32 were available on tissue microarrays (TMAs). Expression analysis of subtype-specific biomarkers, that is, ER, PR, HER-2, Ki-67, CK5/6, and EGFR, was performed on TMAs with IHC. The results show that most *BRCA2 *tumors express luminal markers (24 of 32; 75.0%), of which the majority subcategorize as luminal B (16 of 24; 67%) rather than luminal A (eight of 24; 33.3%) (Table 1). Notably, none of the 32 *BRCA2 *tumors displayed overexpression or high-level amplification of the human epidermal growth factor receptor 2 (*HER2*) oncogene (Table 1). Of the eight hormone-receptor negative *BRCA2 *tumors, all but one was subcategorized as basal-like (seven of eight, 87.5%), of which four displayed EGFR expression, and five were positive for CK5/6 (Table 1).

**Table 1:** Phenotypic characterization of breast tumors derived from *BRCA2 *germline mutation carriers

Biomarkers for definition of phenotypes	0	I-II	III
ER	8 (25%)	7 (22%)	17 (53%)
PR	12 (37%)	14 (44%)	6 (19%)
HER2	22 (69%)	10 (31%)	0 (0)
**Cellular proliferation; The proportion of cells positive for Ki-67**			
Ki-67	**0-14% (1st Qu.)**	**> 14%-35% (IQR)**	**> 35% (3rd Qu.)**
Luminal	8 (33%)	14 (58%)	2 (8%)
Triple-negative	0 (0)	3 (37%)	5 (63%)
			Χ^2 ^test; *P *= 0.004
**Expression of basal features**			
EGFR	**0**	**I**	**≥ II**
Luminal	22 (96%)	1 (4%)	0 (0)
Triple-negative	4 (50%)	1 (13%)	3 (37%)
			Χ^2 ^test; *P *= 0.004
CK5/6	Negative	Positive	
Luminal	24 (100%)	0	
Triple-negative	3 (37%)	5 (63%)	
			Χ^2 ^test; *P *= 0.0003
**pRb/p16 pathway**			
p16 (CDKN2A)	**0**	**I-II**	**III**
Luminal A	3 (38%)	4 (50%)	1 (12%)
Luminal B	9 (60%)	5 (33%)	1 (7%)
Triple-negative	0	1 (12%)	7 (88%)
			Χ^2 ^test; *P *= 0.0007
Cyclin-D1 (CCND1)	**0-15% (1st Qu.)**	**> 15%-75% (IQR)**	**> 75% (3rd Qu.)**
Luminal A	1 (12%)	4 (50%)	3 (38%)
Luminal B	1 (6%)	8 (50%)	7 (44%)
Triple-negative	6 (75%)	2 (25%)	0
			Χ^2 ^test; *P *= 0.005
pRb (RB1)	**0 **(Negative)	**I **(Positive)	
Luminal A	1 (13%)	7 (87%)	
Luminal B	2 (13%)	14 (87%)	
Triple-negative	4 (50%)	4 (50%)	
			Χ^2 ^test; *P *= 0.085
**Morphologic features**			
Histologic grade	**I**	**II**	**III**
Luminal	2 (8%)	11 (44%)	12 (48%)
Triple-negative	1 (12%)	1 (12%)	6 (75%)
			Χ^2 ^test, *P *= 0.27

MKI67 (Ki-67) gene products tightly associate with cellular proliferation and are often used to estimate tumor growth rates [21]. Counting the number of tumor cells positive for Ki-67 against the total number of tumor cells reveals generally higher proliferation rates among triple-negative *BRCA2 *tumors compared with luminal *BRCA2 *tumors (χ^2 ^test; *P *= 0.004; Table 1; Additional File 3). These differences coincide with high p16 (CDKN2A) (χ^2 ^test; *P *= 0.0007), and low pRb (RB1) in the triple-negative subtype of *BRCA2 *mutated breast tumors (χ^2 ^test; *P *= 0.085) (Table 1). Increased p16 expression levels were highly correlated with decreased pRb levels (Spearman correlation rho = -0.63; *P *= 0.0001). Accordingly, low pRb levels coupled with high p16, that is pRb^low^/p16^high^, was commonly found in triple-negative *BRCA2 *tumors (four of eight; 50%), but rarely in luminal *BRCA2 *tumors (two of 23; 8.7%) (χ^2 ^test; *P *= 0.043) (Additional File 3). In contrast, the luminal subtypes of BRCA2 mutated breast tumors were associated with high expression of cyclin-D1 gene products (χ^2^; *P *= 0.005) (Table 1).

### DNA copy-number changes with respect to phenotype in *BRCA2*-associated tumors

**Figure 1:**
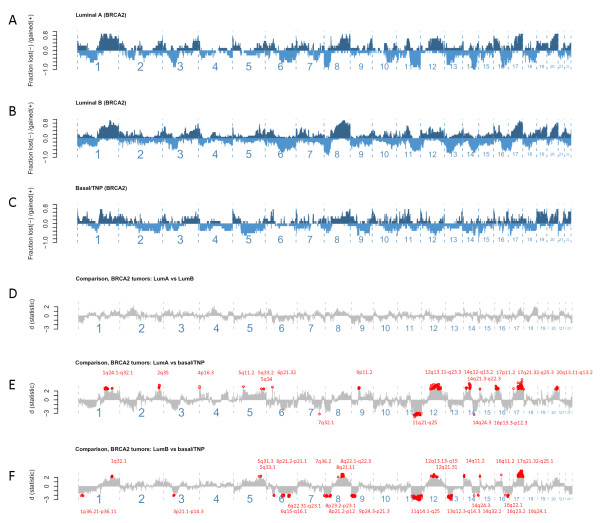
**Event-frequency plots for copy-number alterations and comparison between phenotypic subgroups of *BRCA2 *tumors**. **(a) **The proportion of gains (positive, dark blue) and deletions (negative, light blue) plotted on y-axis for each genomic location ordered from p- to q-arm on x-axis left to right in *BRCA2 *tumors displaying luminal A (*n *= 8), and (b) luminal B (*n *= 16), and **(c) **basal/TNP phenotypes (*n *= 8). **(d) **Comparison in copy-number alterations with significance levels **(d) **shown on y-axis and differences below a false-discovery rate of 0.05 indicated in red (cytoband locations) derived from comparisons between *BRCA2 *tumors displaying luminal A and luminal B phenotypes, **(e) **between *BRCA2 *tumors displaying luminal A and basal/TNP, and **(f) **between *BRCA2 *tumors displaying luminal B and basal/TNP.

Event-frequency plots for copy-number changes in *BRCA2 *tumors displaying luminal A, luminal B, and basal/TNP describe the fraction of tumors within each class, showing gains or losses in order of genomic position (Figure 1a-c). Clearly, certain events occur more frequently in the luminal types of *BRCA2 *tumors compared with those of basal/TNP phenotypes and vice versa. An important observation in this respect is that some changes are inversely altered between the two phenotypic subgroups (that is, luminal and TNP/basal). To illustrate this point, it can be seen that changes over 12 q predominantly occur as gains in the luminal subtypes, whereas they mostly occur as deletions in the basal/TNP subtypes (Figure 1a-c). Detailed comparison between each phenotypic subclass of *BRCA2 *tumors shows that luminal A and B do not differ (Figure 1d). However, substantial differences were revealed between either luminal A or B when compared with TNP/basal types of *BRCA2 *tumors (Figure 1e, f). Some of these differences probably reflect phenotype-related effects independent of *BRCA2 *abnormalities (for example, the events at 5 q and 8 q have been described with respect to phenotype in sporadic tumors elsewhere [10,22]). However, other differences may relate to *BRCA2 *defects; for example, deletions at 13q13.1, covering the *BRCA2 *gene locus, were highly prominent in *BRCA2 *tumors of luminal B types compared with those displaying basal/TNP features (Figure 1f).

**Figure 2:**
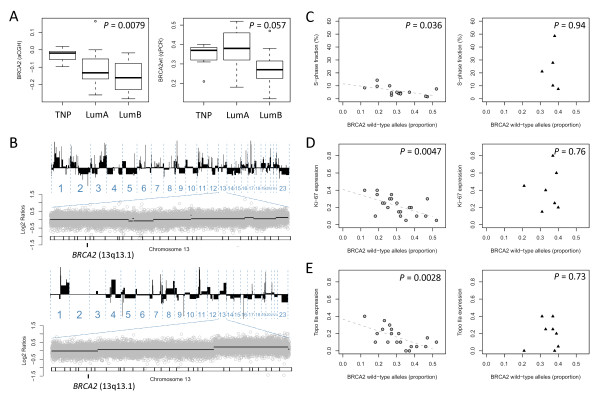
**The effects of *BRCA2 *deletion in phenotypic context**. **(a) **Deletion at the *BRCA2 *locus measured by segmented aCGH log_2 _ratios (left) and TaqMan qPCR (right) in relation to phenotype. **(b) **Example profiles derived from two *BRCA2 *tumors (a19861102, a128049) of the triple-negative subtype analyzed by using 385 K aCGH (about one probe for each 7 kb). DNA gains or deletions are represented on the y-axis as black bars toward positive or negative values, respectively, in order of the chromosomal location from p- to q-arm (left to right). Focused views of chromosome 13 are displayed below each of the two profiles showing log_2 _ratios (gray dots) on y-axis by nucleotide position on x-axis from p-arm to q-arm (left to right) with cytobands and the *BRCA2 *gene locus at 13q13.1 indicated as shown at the bottom of each plot. **(c) **S phase, **(d) **Ki-67, and **(e) **Topo IIa in relation to changes at the *BRCA2 *gene locus, affecting the wild-type allele, in luminal- (left panel, gray circles) and triple-negative phenotypes (right panel, black triangles).

Given the importance of the *BRCA2 *locus in this study, we specifically explored the segmented aCGH log_2 _ratios covering the *BRCA2 *gene along with analysis of allele-specific *BRCA2 *loss with qPCR analysis. These methods showed strong concordance (that is, changes in aCGH log_2 _ratios at the *BRCA2 *locus significantly correlate with estimates of *BRCA2 *wild-type allele proportions by qPCR; Pearson correlation *r *= 0.69; *P *= 0.000025). Looking separately at each of the two *BRCA2 *phenotypic subgroups (luminal and basal/TNP), a clear trend toward deletion at the *BRCA2 *locus emerged for the luminal types of *BRCA2 *tumors, in particular, in the more-proliferative luminal B type (Figure 2a). Two individual cancer genomes analyzed by aCGH are shown to illustrate lack of deletion events affecting the *BRCA2 *gene locus at 13q13.1 in the TNP subtype of *BRCA2 *tumors (Figure 2b). To examine this further, we studied the relation on a continuous scale with markers of cellular proliferation that are S phase by flow cytometry, along with expression of proliferation-associated genes measured on a semicontinuous scale that is, Ki-67, and Topo IIα. Estimates for S-phase fractions, indicative of cells undergoing DNA replication, were found to be associated with decreased proportions of *BRCA2 *wild-type alleles in *BRCA2 *tumors of the luminal phenotype (Pearson correlation, *r *= -0.56; *P *= 0.036) (Figure 2c). This relation was not found in *BRCA2 *tumors displaying basal/TNP features, wherein very high proliferation rates are attained independent of deletion events over the *BRCA2 *locus at 13q13.1. Expression of Ki-67, as a measure of cellular proliferation, has the benefit of enabling stromal cells to be ignored, which is more difficult in analysis of S phase with flow cytometry. Estimates for the proportion of Ki-67-positive tumors cells were found to be correlated with decreased proportions of wild-type *BRCA2 *alleles in *BRCA2 *tumors displaying luminal characteristics (Pearson correlation, *r *= -0.59; *P *= 0.0047), whereas the same was not found in *BRCA2 *tumors displaying basal/TNP phenotype (Pearson correlation, *r *= 0.13, *P *= 0.76) (Figure 2d). In support for the preceding, the same associations, with respect to *BRCA2 *wild-type allele loss and phenotype, were identified for estimates of the cell-cycle associated gene product Topo IIα **(**Figure 2e).

### Genetic networks

**Figure 3:**
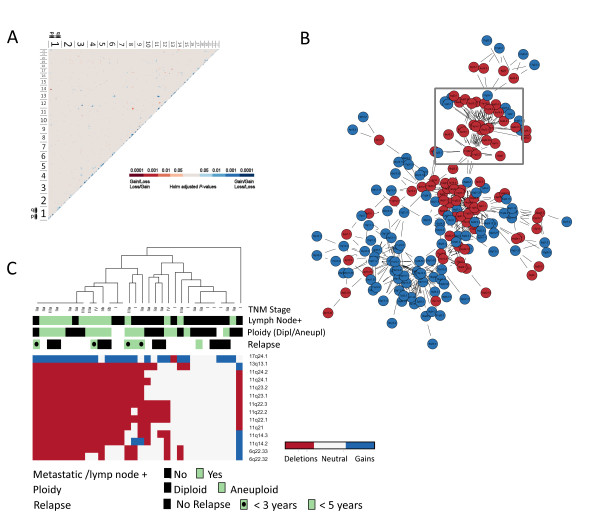
**Genetic networks in *BRCA2 *tumors**. **(a) **Matrix of associations with blue and red reflecting *P *values (Holm adjustment for multiple testing) for distinct chromosomal regions where copy-number changes co-occur in patterns of loss-loss or gain-gain relations, represented in blue, or gain-loss relations represented in red. **(b) **Genetic networks; interchromosomal links constructed for relations below Holm-adjusted *P *values of 0.001 (edges) between distinct genomic regions (nodes), with deletions in red and gains in blue, showing multiple links adjacent to the *BRCA2 *locus at 13q13.1 (box). **(c) **Hierarchic cluster analysis of copy-number changes, deletions in red and gains in blue, over the subnet of chromosomal regions linked to 13q13.1 (*BRCA2*) in relation to the occurrence of lymph node metastasis at diagnosis, and later relapse involving distant metastases represented in colors of green (positive) or black (negative), ploidy as green for diploid and black for aneuploid (see color codes at bottom of heatmap), along with TNM stage.

DNA copy-number changes showing significant associations between distinct chromosomal regions in the set of 33 *BRCA2 *tumors were identified, and visualized as an undirected node-and-edge graph in which significant correlations are represented as edges, with genomic regions as nodes (Figure 3a, b; Additional File 4). This analysis identified a set of 14 cytobands involving three distinct interchromosomal links in association with the *BRCA2 *locus at 13q13.1, that is, deletions at 6q22.32-q22.33 and 11q14.2-q24.1, along with copy-number gains at 17q24.1 (Figure 3b, Additional File 4). It is important to note here that the data on *BRCA2wt*-specific loss confirmed the links identified with respect to 6q22.32-q22.33 and 11q14.2-q24.1 (Additional File 5). Based on the *BRCA2*-linked networking regions (6q22.32-q22.33, 11q14.2-q24.1, 13q13.1, and 17q24.1), two subgroups were identified through cluster analysis (Figure 3c). Evidently, these subgroups differ by the degree of change over the regions identified in association with the *BRCA2 *gene locus. Here, measures of higher clinical staging at the time of diagnosis, involving metastasis to the lymph nodes, were more prominent in the subgroup showing changes over the *BRCA2*-linked networking regions (Figure 3c). Data on patient relapse were available in some of these cases, defined as an instance of subsequent metastasis or recurrence, which provided support for the hypothesis generated here that DNA copy-number changes over the *BRCA2*-linked networking regions promote progression toward more-advanced forms of the disease (see Figure 3c). In all cases, metastasis to more-distant sites of the body was involved, of which all but one occurred as bone metastases. To determine whether the network of genetic changes identified in relation to the *BRCA2 *locus at 13q13.1 is specific for *BRCA2 *mutated breast cancers, we looked at our previously published data on sporadic breast cancers [15]. As expected, the *BRCA2 *locus at 13q13.1 was not represented within the genetic networks constructed by using the same approach for sporadic breast cancers (Additional File 4).

**Figure 4:**
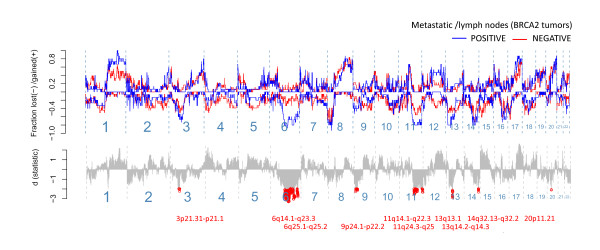
**Two-class comparative analysis displaying event-frequency plots for *BRCA2 *tumors with (blue) and without (red) evidence for lymph node metastases at diagnosis**. The fraction of tumors with acquired changes are represented on the y-axis with positive values for gains, and negative for deletions, ordered by chromosomal location on x-axis from p- to q-arm, from left to right. The significance scores (d), derived from a permutation-based procedure (SAM two-class unpaired), representing the strength of association, are shown in the lower panel with relations below a false-discovery rate of 0.05 indicated in red (cytoband locations).

In a two-class comparison for *BRCA2 *tumors with or without evidence of metastasis to the lymph nodes, or other more distant sites, loss over the *BRCA2 *locus at 13q13.1 was identified in this analysis as significantly associated with metastasis to the lymph nodes, along with the *BRCA2*-linked networking regions at 6 q and 11 q (Figure 4). Of these, deletions at chromosome 6 q were the most predictive for lymph node positivity, or "metastatic capacity," with the strongest associations (significance peaks) found at 6q16.3 and 6q22.33. Looking at the frequency of copy-number changes at 6q22.33, we identified a shift toward increased frequency of deletions at a location close to the *PTPRK *gene, which affected four other genes, that is *EEF1DP5*, *LAMA2*, *ARHGAP18*, and *C6orf191 *(Additional File 6). Fine-tiling aCGH analysis at exon-level resolution (about one probe for each 400 bp) covering chromosome 6 confirmed deletions at 6q22.33 (Additional File 7). This analysis shows that the events at 6q-arm occur as complex deletions confined within the 6 q chromosomal region. Here, one of the tumors had acquired a breakpoint event located within the 6q22.33 cytoband proximal to the *PTPRK *gene affecting upstream regions (Additional File 7). This subregion within the 6q22.33 cytoband was not affected in the lymph-node-negative tumor, but rather involved a region downstream of the *PTPRK *gene (Additional File 7).

### The analysis of ploidy in *BRCA2 *mutated breast cancers with respect to DNA copy-number changes by aCGH

Data available on ploidy were analyzed with respect to DNA copy-number changes to follow-up on recent results demonstrating poor disease outcome for *BRCA2 *carriers diagnosed with breast tumors of the diploid type (Tryggvadottir *et al.*, 2011; unpublished data). In line with the less-favorable prognosis associated with the diploid type of *BRCA2 *tumors, we found that *BRCA2 *mutated tumors, having acquired changes over the *BRCA2*-networking regions, were often identified as diploid with flow cytometry (Figure 3c).

**Figure 5:**
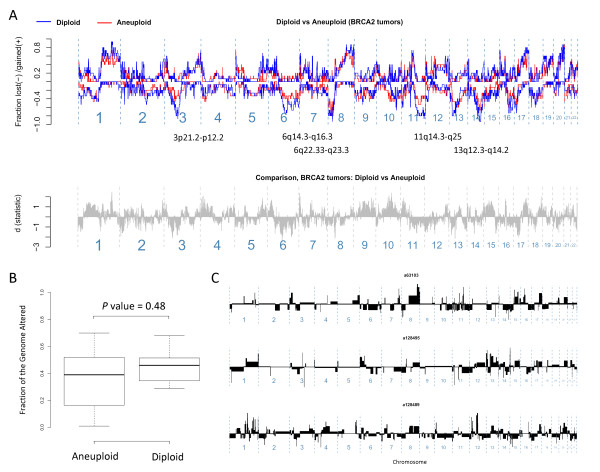
**The effects of ploidy on DNA copy-number changes in *BRCA2 *breast tumors**. **(a) **Event-frequency plots to compare the genomic regions affected in diploid (blue) and aneuploid (red) types of *BRCA2 *tumors. **(b) **The proportion of the genome altered in *BRCA2 *tumors with respect to ploidy. **(c) **Three example genomic profiles analyzed by aCGH to illustrate complex changes in *BRCA2 *tumors classified as diploid by flow-cytometry. The genomic profiles represent DNA copy-changes number toward gains (above) or losses (below) on the y-axis by chromosomal position on the x-axis from p- to q-arms, left to right, respectively.

To study this further, we compared event-frequency plots and the altered genome fractions for *BRCA2 *tumors of the diploid and aneuploid types. This analysis shows that the amounts of genomic changes are not fewer in diploids (Figure 5a) and, although not statistically significant, that the regions affected more frequently by DNA copy-number changes in the diploid types overlap with those referred to here as the *BRCA2*-networking regions (Figure 5b). It is clear that the genomes of *BRCA2 *mutated breast cancers classified as diploid are extensively altered, and individual examples are presented here to demonstrate complex patterns of genomic changes (Figure 5c).

## Discussion

In this study, we show that breast tumors from *BRCA2 *999del5 mutation carriers, displaying luminal- or basal/triple-negative phenotypes (TNPs), differ with respect to patterns of copy-number changes and markers of pRb/p16 pathway dysfunction, suggesting divergent paths of tumor evolution. This finding led to important observations regarding loss of the wild-type (wt) *BRCA2 *allele in luminal types of *BRCA2 *tumors, where it associates with increased tumor growth rates. Furthermore, we find that deletion of the *BRCA2*wt allele represents a part of a larger network of co-occurring genetic changes that relate to advanced progression, involving deletions at 6q22.32-q22.33, 11q14.2-q24.1, as well as gains at 17q24.1. These results suggest that loss of the *BRCA2*wt allele is not required in tumor initiation, but rather plays an important role later in disease progression. Recently, it was demonstrated that heterozygosity for *Brca2 *is sufficient to promote carcinogenesis in a murine model of familial pancreatic cancer and also that LOH might not be essential for human pancreatic ductal carcinogenesis in *BRCA2 999del5 *mutation carriers [7]. In addition, an earlier study on breast cancer samples from carriers of different *BRCA2 *mutations showed retention of the *BRCA2wt *allele in some cases, providing support for our conclusion [8]. We conclude that loss of the *BRCA2wt *allele, although not required in tumor initiation, is not a randomly occurring passenger but rather an important driver event in leading to selective growth advantages and higher clinical staging of the disease. These effects are likely indirect, resulting from accelerated mutation rates, from which advantageous mutations can arise and lead to a more aggressive phenotype.

The majority of the *BRCA2*-associated tumors under study displayed luminal characteristics, whereas the remaining part was of basal/triple-negative phenotype (TNP). Notably, none of the *BRCA2 *tumors showed overexpression or high-level amplification of the *HER-2 *oncogene. This subdivision of *BRCA2 *tumors into phenotypic subgroups is in agreement with our previous study and those of others [14,15]. The loss of pRb observed in the basal/TNP subtype of *BRCA2 *mutated breast tumors likely reflects acquired defects in the *RB1 *gene. Indeed, high expression of CDKN2A (p16) gene products, indicative of functional defects in *RB1*, has been described as a defining feature of basal-like breast cancers, and it has been suggested that this may represent an important early event in bypassing cellular stress and replicative senescence [23,24]. The negative-feedback loop between high p16 and low pRb has been described and is known to be the result of pRb binding to the CDKN2A (p16) gene promoter, leading to transcriptional repression of CDKN2A [25]. Increased expression of CDKN2A (p16) gene products arises under normal circumstances as a response to stress signals, such as telomere crisis or DNA damage. Thus, unimpeded cellular proliferation, indicated by expression of cell-cycle markers such as Ki-67, in combination with the presence of high p16 expression levels, could be indicative of abrogated p16/RB checkpoint control [23]. We found high expression of MKI67 (Ki-67) gene products, a marker for cellular proliferation, in basal/TNP *BRCA2 *tumors, together with low pRb and high p16 levels. In contrast, the luminal subgroup of *BRCA2 *tumors rarely showed high p16 expression levels and almost invariably showed pRb expression. Given the association for loss of *BRCA2*wt alleles with respect to the luminal phenotype, this finding would have been unexpected as the *RB1 *(*pRb*) gene resides close to *BRCA2 *on chromosome 13q. Nevertheless, the association of p16/pRb dysfunction to the basal/TNP subtype, identified here in *BRCA2 *mutated breast tumors, is similar to that reported in sporadic tumors suggesting that the events leading to the basal/TNP phenotype in carriers and noncarriers could be the same. In this respect, it has been demonstrated that *BRCA1 *inactivation in sporadic disease leads to development of basal/TNP breast cancers [26,27]. In our recent report, we described the loss of pRb, coupled with high p16, in *BRCA1*-defective breast cancers [27]. We therefore postulate that the event of acquired defects in the *BRCA1 *gene could have the same effect in *BRCA2 *mutation carriers, as seen in sporadic breast cancers. Presumably, this would alleviate selection pressure for loss of the *BRCA2*wt allele in *BRCA2 *mutation carriers. This is consistent with the data presented here, demonstrating lack of definitive *BRCA2*wt loss in the basal/TNP subclass of *BRCA2 *tumors.

Whole-genome copy-number changes in this same set of *BRCA2 *tumors analyzed with high-resolution (~7 kbp) aCGH demonstrated substantial differences between luminal- and basal/TNP subtypes of *BRCA2 *tumors. These differences involved frequent alterations at multiple chromosome locations in luminal *BRCA2 *tumors that were infrequently or inversely altered in basal/TNP *BRCA2 *tumors. Their unique patterns of copy-number changes suggest that the two phenotypic subclasses of *BRCA2 *tumors develop in different ways. Of particular interest here are the luminal subtypes of *BRCA2 *tumors showing more-frequent deletions over the *BRCA2 *gene locus. Here, gradual decreases in the proportion of *BRCA2wt *alleles present within the tumor mass were correlated with increased growth rates, suggesting selective advantages for the event of *BRCA2wt *loss. It can be hypothesized that this relation reflects a progression pattern involving clonal expansion of tumor cells that have acquired deletion of the *BRCA2*wt allele. This appears contradictory to previous studies demonstrating that targeted disruption of the *Brca2 *gene negatively affects cell-cycle progression [28]. Other changes could therefore be required to overcome the deleterious effects of *BRCA2 *deficiency. In this respect, the network of genetic changes affecting 6q22.32-q22.33, 11q14.2-q24.1, and 17q24.1 identified in association with *BRCA2wt *loss could be of critical importance in promoting survival of *BRCA2*-defective tumor cells. This would give rise to cancer cells with *BRCA2 *deficiency, thereby leading to accelerated mutation rates and, through the forces of natural selection, advantageous changes could accumulate and eventually lead to disease progression. Indeed, we find that the set of co-occurring changes linked to the *BRCA2 *region at 13q.13.1 coincide with those that associate with advanced disease progression involving metastasis to the nodes, and patient relapse involving distant metastases. Here, deletion events at chromosome 6 q were the strongest predictors of metastatic capacity. To gain further insight into the genetic events occurring at this location, we performed fine-tiling aCGH analysis (exon-level resolution). Detailed views of changes at 6q22.33 (that is, the highest ranking of the *BRCA2*-linked genomic regions) suggested the involvement of genes located at, or downstream, of a breakpoint event disrupting protein tyrosine phosphatase, receptor type, K (*PTPRK*), a putative tumor suppressor gene known to be involved in TGF-β signaling [29]. Interestingly, the 6q22.33 region has been identified as a breast cancer susceptibility locus where two candidate genes are located, that is, RING finger protein 146 (*RNF146*) and enoyl coenzyme A hydratase domain-containing 1 (*ECHDC1*) [30]. Looking at breakpoint events at chromosome 6 q by using the fine-tiling aCGH analysis, we did not find strong evidence for gross genetic changes indicative of error-prone DNA repair through NHEJ, similar to that reported for the *PTEN *gene at chromosome 10 q in *BRCA1 *mutated breast tumors [31]. However, it is clear that, in most cases, these events involve complex changes as they are not characterized by loss of the entire 6 q arm. Deletions at 11 q have been described previously in association with *BRCA2 *breast tumors wherein aberrant expression of *ATM *has been demonstrated [32]. However, the region at 11q14.2-q22.2, including *MRE11*, has not been studied previously in this respect. The subnet of genetic links associated with the *BRCA2 *gene locus warrants further investigation in terms of which genes are involved and whether they explain disease outcome in *BRCA2 *carriers and other patients with or without a family history of the disease. This could be of potential significance in defining a set of biomarkers for use in clinical settings to identify HR defects (that is, markers of BRCAness) [33].

In looking at data from flow-cytometry, we observed diploidy as a common feature of those *BRCA2 *tumors displaying changes over the *BRCA2*-linked regions. Clearly, this was unexpected, as diploid tumors are usually associated with genomes characterized by few or no genomic changes (that is, simple genomes) [34]. This finding has relevance to a recent study on survival in *BRCA2 *mutation carriers, wherein tumors classified as diploid were found to be associated with fast disease progression independent of hormone receptors, histologic grade, and TNM stage (Tryggvadottir *et al.*, 2011, unpublished data). Here, we add important insight into this, with data demonstrating complex genetic changes in genomes of *BRCA2 *tumors identified as diploid with flow-cytometry. The presence of genomic complexity in *BRCA2 *tumors of the diploid class demonstrates that they are pseudo-diploids. Nevertheless, the differences in disease outcome with respect to ploidy in *BRCA2 *carriers are compelling and suggestive of divergent tumor-progression paths. In this respect, it has been shown that approximately 30% of all breast tumors classified as diploid by flow-cytometry have acquired complex patterns of genetic changes, as identified through aCGH-based method (ROMA) [34]. Importantly, the two different instability-types described (that is, firestorm and sawtooth), were associated with poor disease outcome. Thus, it seems likely that at least a certain proportion of tumors classified as diploid by conventional flow-cytometry could be promoted by acquired defects in components of the HR machinery, possibly involving the *BRCA2 *gene.

Inhibitors of PARP1 gene products have been shown to kill *BRCA1- *or *BRCA2*-defective cells selectively, with no effects on heterozygous cells, and such inhibitors are currently being tested in clinical trials [35]. Our results could have clinical implications regarding the use of PARP inhibitors, with observations demonstrating late involvement for loss of the *BRCA2*wt allele. In this respect, PARP inhibitors will probably not be sufficient alone (that is, as monotherapy), for treatment of *BRCA2 *carriers. Nevertheless, the risk of progression toward advanced disease in the event of *BRCA2*wt allele loss is of considerable concern, and our results therefore strongly support adjuvant use of PARP inhibitors in standard treatment of all *BRCA2 *mutation carriers.

## Conclusions

In conclusion, our results demonstrate phenotypic heterogeneity of breast cancers arising in carriers of the same *999del5 BRCA2 *mutation. We show that the luminal- and basal/TN phenotypes develop through divergent tumor evolutionary paths. Here, loss of the *BRCA2 *wild-type allele occurs more frequently in the luminal subtype, where it correlates with increased tumor growth rates. Furthermore, the event of *BRCA2*wt allele loss occurs in the context of other genetic changes, involving deletions at 6 q, 11 q, and copy-number gains at 17 q. These changes, including deletions over the *BRCA2 *gene locus, were found to be associated with higher clinical staging at diagnosis involving metastasis to the lymph nodes, and correlated with patient relapse. These observations could explain why *BRCA2 *carriers have predilection for developing breast cancers of the luminal B phenotype. The findings presented here, suggesting that loss of the *BRCA2*wt allele is a late, rather than early, event in progression toward metastatic disease, have clinical relevance supporting adjuvant, and not single-agent, use of PARP inhibitors in treatment of *BRCA2 *carriers.

## Abbreviations

CGH: comparative genomic hybridization; DSB: double-stranded break; HR: homologous recombination; IHC: immunohistochemistry; LumA: Luminal-A; LumB: Luminal-B; NHEJ: nonhomologous end joining; SSA: single-strand annealing; TMA: tissue microarrays; TNP: triple-negative phenotype.

## Competing interests

The authors declare that they have no competing interests.

## Authors' contributions

OAS contributed to the study design and performed the aCGH analysis along with statistical analysis, data mining, and writing of the manuscript. OAS and JGJ selected tumor tissue for TMAs. KO constructed the TMAs and performed IHC analysis. OAS and JGJ scored the IHC results. JGJ scored histologic grade and TNM staging. OTJ contributed clinical data, and SV contributed to the aCGH analysis. HB and SKB performed the TaqMan *BRCA2 *allele-specific quantitative PCR (qPCR). JEE conceived of the study and was in charge of its design and the coordination and writing of the manuscript. All authors read and approved of the manuscript.

## Supplementary Material

Additional file 1 **Phenotype and clinical parameters**. Table listing tumor phenotype and clinical parameters including breast cancer subtype, histologic grade, and TNM stage. Click here for file

Additional file 2 **Antibodies for expression analysis on TMAs**. Table listing the antibodies used for immunohistochemistry on tissue microarrays (TMAs). Click here for file

Additional file 3 **The expression of Ki-67, p16, and pRb in *BRCA2 *mutated breast cancers by phenotype**. Expression of Ki-67, p16, and pRb by IHC on tissue microarrays (TMAs) in subtypes of *BRCA2 *breast tumors. **(a) **The distribution of Ki-67 expression levels, reflecting cellular proliferation, compared between BRCA2 tumors of basal/triple-negative and luminal phenotypes. Example IHC analysis for Ki-67 shows higher proportion of positive cells in *BRCA2 *tumors of triple-negative (lower panel) compared with luminal phenotypes (upper panel). **(b) **Expression of p16 (CDKN2A) in *BRCA2 *tumors by phenotype with low expression of pRb (IHC null) indicated, see Table 1 for details. Examples show representative *BRCA2 *tumors of luminal- (upper panel) and triple-negative (lower panel) phenotypes. Click here for file

Additional file 4 **The complete set of links identified in breast cancers graphically represented as node-and-edge graphs, shown here separately for those derived from *BRCA2 *carriers and sporadic cases**. Genetic networks constructed for relations below Holm-adjusted *P *values of 0.001 (edges) between distinct genomic regions (nodes), with deletions in red and gains in blue. The links identified, reflecting co-occurring genetic changes, were split into sets of inter- and intrachromosomal links characterized as those involving distinct chromosomes (inter, left on figure) or distinct arms on the same chromosome (intra, right on figure), respectively. **(a) **The genetic networks constructed for *BRCA2 *mutated breast cancers, and **(b) **those arising in the sporadic setting. Click here for file

Additional file 5 ***BRCA2 *wild-type allele specific qPCR examined with respect to DNA copy-number changes**. Deletion of wild-type *BRCA2 *alleles by TaqMan qPCR analysis were examined on a continuous scale in relation with the genome-wide segmented aCGH log_2 _ratios by using a regression model with repeated permutations to address the problem of multiple testing, as implemented in SAM (quantitative analysis). The significance scores (r) derived from this analysis are plotted on the y-axis for each genomic location ordered from p- to q-arm on the x-axis, from left to right, with relations below a false-discovery rate of 0.0001 indicated by red dots. Click here for file

Additional file 6 **Detailed analysis of event frequencies at chromosome 6 q in *BRCA2 *mutated breast cancers**. Analysis of DNA copy-number changes occurring at chromosome 6 in BRCA2 tumors. **(a) **Top: Comparison for the frequency of events in BRCA2 tumors with and without evidence for metastatic spread to the lymph nodes or other sites at diagnosis, a trait reflecting metastatic capacity, represented in red and orange, respectively (see legend at the top right corner). Bottom: Zooming in on the frequency of events at 6q22.33, selected for further analysis, as this region strongly associates with metastatic capacity in *BRCA2 *tumors while also being linked to deletions over the *BRCA2 *gene locus at 13q13.1. **(b) **Top: Example of fine-tiling aCGH analysis (exon-level resolution) for chromosome 6 in one *BRCA2 *breast tumor where aCGH log_2 _ratios are plotted on the y-axis in order of nucleotide position on the x-axis. Dashed vertical lines represent locations of cytoband boundaries, as indicated below, and solid horizontal red lines represent output derived from CBS analysis for detecting copy-number changes with negative values of segmented aCGH log_2 _ratios reflecting change toward deletions, and positive values as change toward copy-number gains. Bottom: Enlarged, detailed view of the entire 6q22.33 region in this tumor shows a breakpoint event occurring nearby the *PTPRK *gene, a frequent site of deletion events in metastatic *BRCA2 *tumors (see data in Figure 5a, bottom panel) affecting four other genes within this cytoband (*EEF1DP5, LAMA2, ARHGAP18*, and *C6orf191*). Click here for file

Additional file 7 **aCGH analysis using fine-tiling arrays (400 bp) for chromosome 6 in BRCA2 mutated breast cancers**. Fine-tiling aCGH analysis (exon-level resolution) covering chromosome 6, with about one probe for each 400 bp, was performed to reanalyze and confirm events at this chromosome with greater clarity in three of the *BRCA2 *tumors. The aCGH log_2 _ratios are plotted on the y-axis in order of nucleotide position on the x-axis, with dashed vertical lines representing locations of cytoband boundaries, as indicated below. Solid horizontal red lines represent output derived from CBS analysis for detecting copy-number changes with negative values of segmented aCGH log_2 _ratios reflecting change toward deletions, and positive values as change toward copy-number gains. Click here for file
