# Supplementary figures and images for: Genomic and phenotypic analysis of BRCA2 mutated breast cancers reveals co-occurring changes linked to progression
Source: Breast Cancer Res. 2011 Sep 29;13(5):R95. doi: 10.1186/bcr3020 (PMC3262207; doi:10.1186/bcr3020)

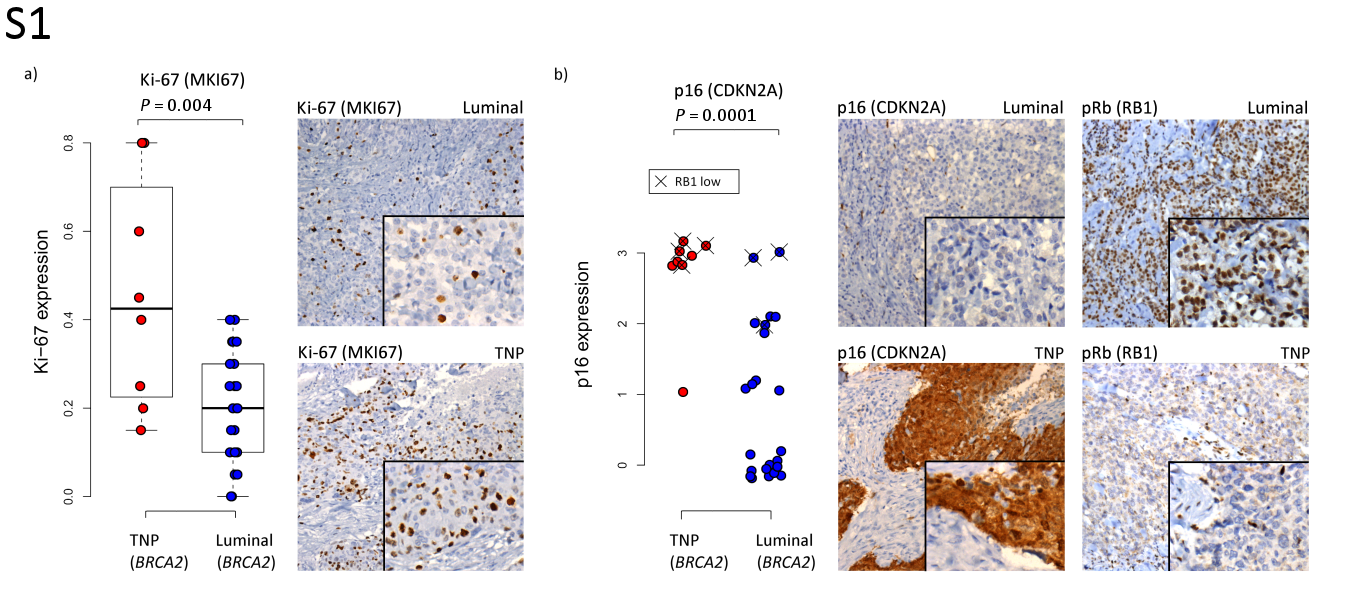

Supplement: Additional file 3 — The expression of Ki-67, p16, and pRb in BRCA2 mutated breast cancers by phenotype. Expression of Ki-67, p16, and pRb by IHC on tissue microarrays (TMAs) in subtypes of BRCA2 breast tumors. (a) The distribution of Ki-67 expression levels, reflecting cellular proliferation, compared between BRCA2 tumors of basal/triple-negative and luminal phenotypes. Example IHC analysis for Ki-67 shows higher proportion of positive cells in BRCA2 tumors of triple-negative (lower panel) compared with luminal phenotypes (upper panel). (b) Expression of p16 (CDKN2A) in BRCA2 tumors by phenotype with low expression of pRb (IHC null) indicated, see Table 1 for details. Examples show representative BRCA2 tumors of luminal- (upper panel) and triple-negative (lower panel) phenotypes. [file bcr3020-S3.TIFF]

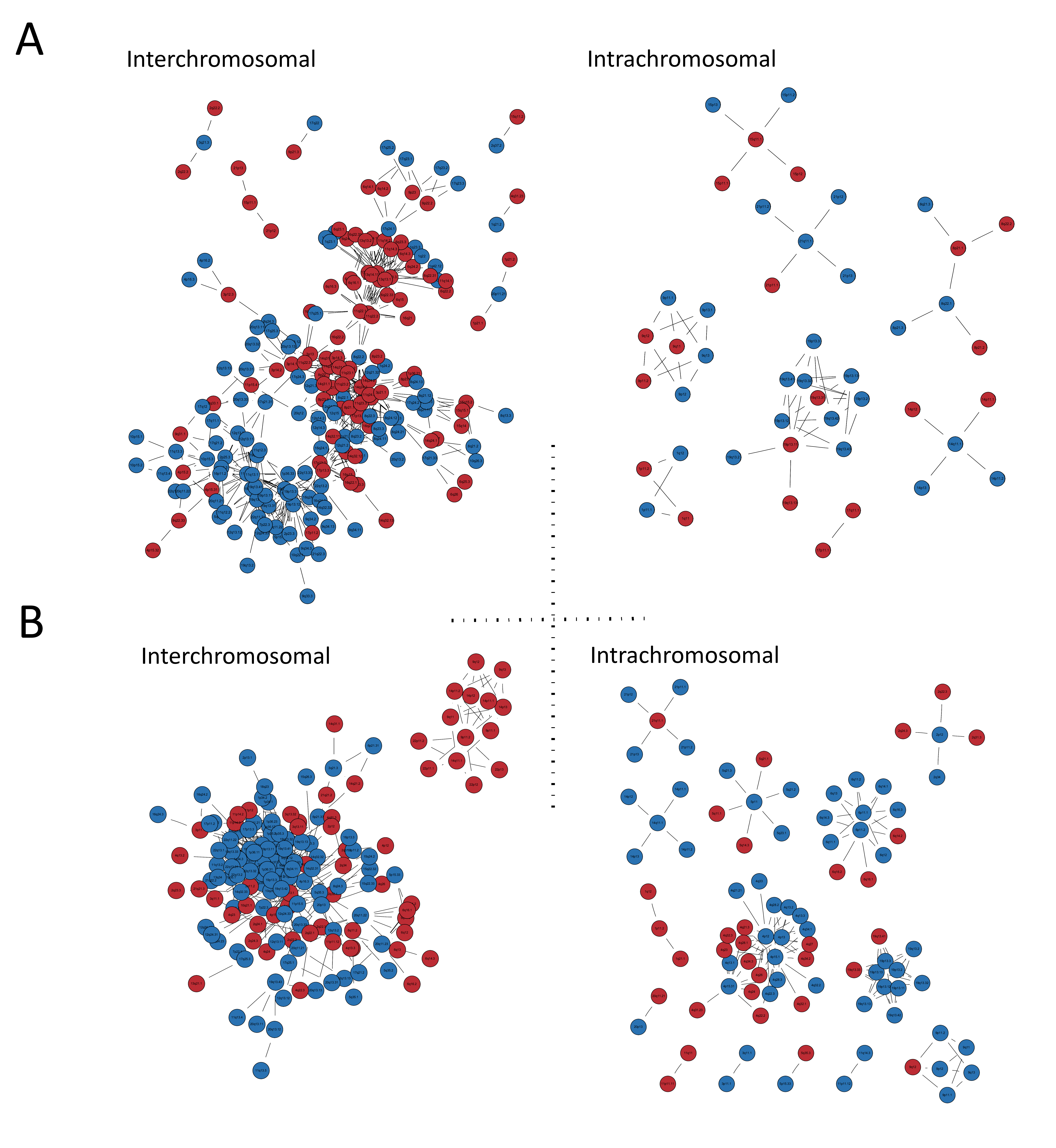

Supplement: Additional file 4 — The complete set of links identified in breast cancers graphically represented as node-and-edge graphs, shown here separately for those derived from BRCA2 carriers and sporadic cases. Genetic networks constructed for relations below Holm-adjusted P values of 0.001 (edges) between distinct genomic regions (nodes), with deletions in red and gains in blue. The links identified, reflecting co-occurring genetic changes, were split into sets of inter- and intrachromosomal links characterized as those involving distinct chromosomes (inter, left on figure) or distinct arms on the same chromosome (intra, right on figure), respectively. (a) The genetic networks constructed for BRCA2 mutated breast cancers, and (b) those arising in the sporadic setting. [file bcr3020-S4.TIFF]

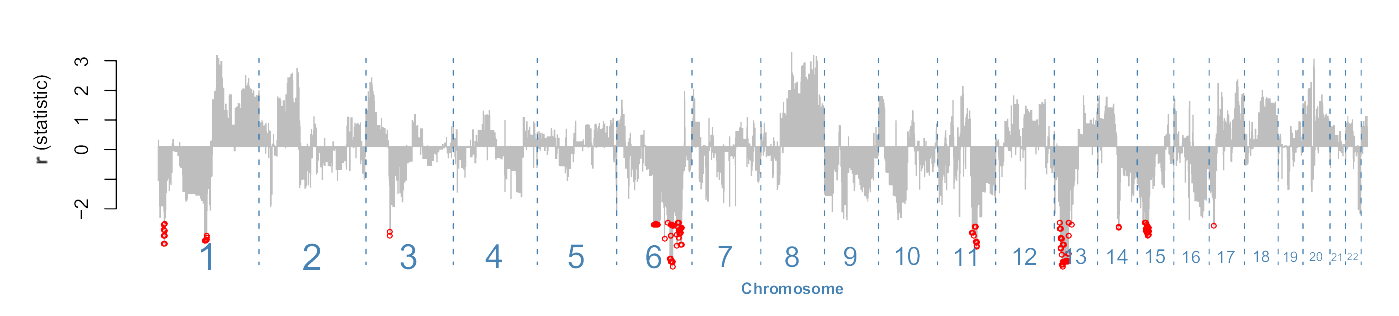

Supplement: Additional file 5 — BRCA2 wild-type allele specific qPCR examined with respect to DNA copy-number changes. Deletion of wild-type BRCA2 alleles by TaqMan qPCR analysis were examined on a continuous scale in relation with the genome-wide segmented aCGH log2 ratios by using a regression model with repeated permutations to address the problem of multiple testing, as implemented in SAM (quantitative analysis). The significance scores (r) derived from this analysis are plotted on the y-axis for each genomic location ordered from p- to q-arm on the x-axis, from left to right, with relations below a false-discovery rate of 0.0001 indicated by red dots. [file bcr3020-S5.TIFF]

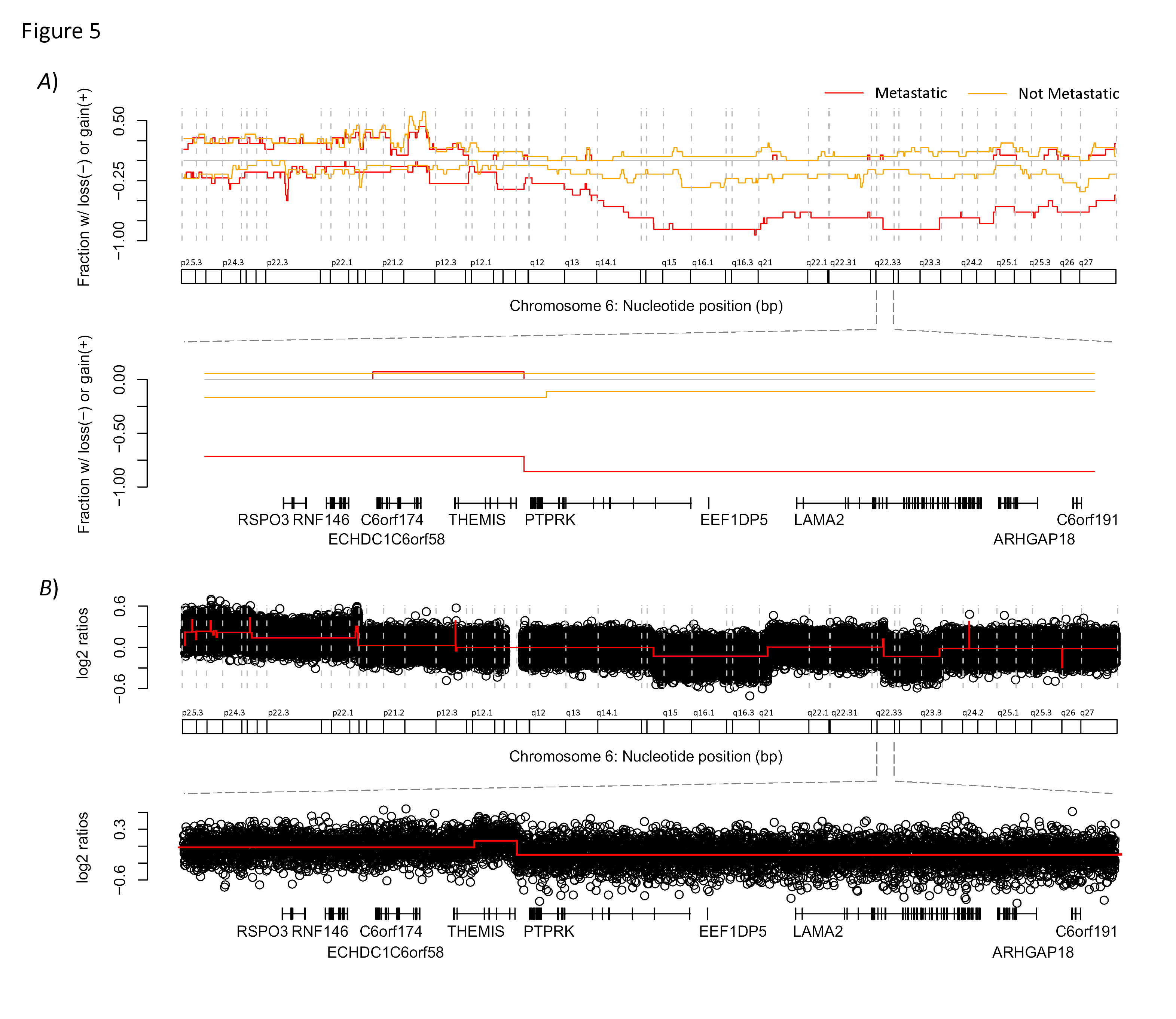

Supplement: Additional file 6 — Detailed analysis of event frequencies at chromosome 6 q in BRCA2 mutated breast cancers. Analysis of DNA copy-number changes occurring at chromosome 6 in BRCA2 tumors. (a) Top: Comparison for the frequency of events in BRCA2 tumors with and without evidence for metastatic spread to the lymph nodes or other sites at diagnosis, a trait reflecting metastatic capacity, represented in red and orange, respectively (see legend at the top right corner). Bottom: Zooming in on the frequency of events at 6q22.33, selected for further analysis, as this region strongly associates with metastatic capacity in BRCA2 tumors while also being linked to deletions over the BRCA2 gene locus at 13q13.1. (b) Top: Example of fine-tiling aCGH analysis (exon-level resolution) for chromosome 6 in one BRCA2 breast tumor where aCGH log2 ratios are plotted on the y-axis in order of nucleotide position on the x-axis. Dashed vertical lines represent locations of cytoband boundaries, as indicated below, and solid horizontal red lines represent output derived from CBS analysis for detecting copy-number changes with negative values of segmented aCGH log2 ratios reflecting change toward deletions, and positive values as change toward copy-number gains. Bottom: Enlarged, detailed view of the entire 6q22.33 region in this tumor shows a breakpoint event occurring nearby the PTPRK gene, a frequent site of deletion events in metastatic BRCA2 tumors (see data in Figure 5a, bottom panel) affecting four other genes within this cytoband (EEF1DP5, LAMA2, ARHGAP18, and C6orf191). [file bcr3020-S6.TIFF]

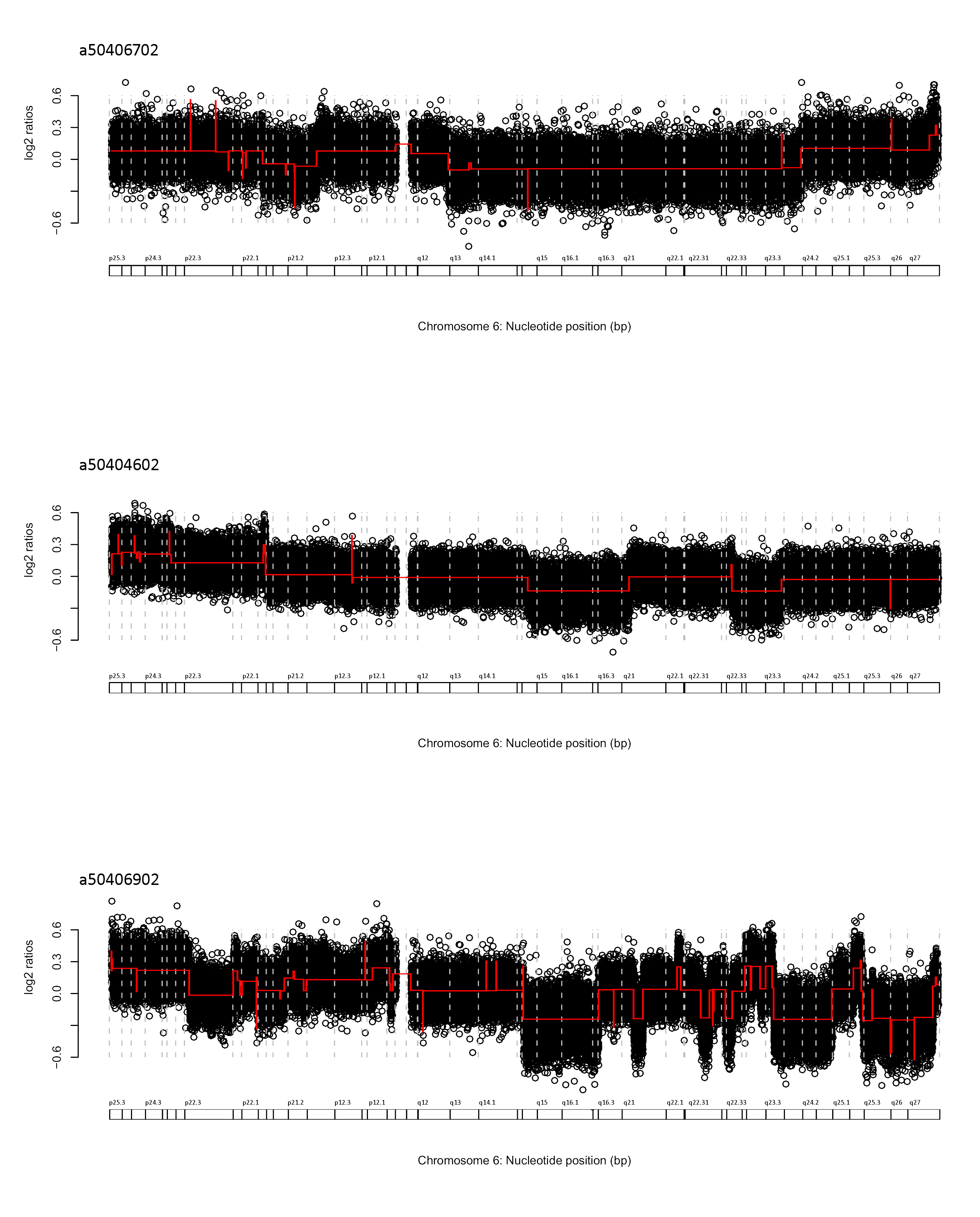

Supplement: Additional file 7 — aCGH analysis using fine-tiling arrays (400 bp) for chromosome 6 in BRCA2 mutated breast cancers. Fine-tiling aCGH analysis (exon-level resolution) covering chromosome 6, with about one probe for each 400 bp, was performed to reanalyze and confirm events at this chromosome with greater clarity in three of the BRCA2 tumors. The aCGH log2 ratios are plotted on the y-axis in order of nucleotide position on the x-axis, with dashed vertical lines representing locations of cytoband boundaries, as indicated below. Solid horizontal red lines represent output derived from CBS analysis for detecting copy-number changes with negative values of segmented aCGH log2 ratios reflecting change toward deletions, and positive values as change toward copy-number gains. [file bcr3020-S7.TIFF]
